# Regulatory T cells-centered regulatory networks of skeletal muscle inflammation and regeneration

**DOI:** 10.1186/s13578-022-00847-x

**Published:** 2022-07-22

**Authors:** Ziyu Chen, HaiQiang Lan, ZhaoHong Liao, JingWen Huang, XiaoTing Jian, Jijie Hu, Hua Liao

**Affiliations:** 1 Guangdong Provincial Key Laboratory of Construction and Detection in Tissue Engineering; Department of Anatomy, School of Basic Medical Science, Southern Medical University, Guangzhou, 510515 China; 2 Clinical Medicine in 8-Year Program, Southern Medical University, Guangzhou, 510515 China; 3 Department of Orthopaedics and Traumatology, Nanfang Hospital, Southern Medical University, Guangzhou, 510515 China; 4 Guangdong Provincial Key Laboratory of Construction and Detection in Tissue Engineering, Department of Anatomy, School of Basic Medical Science, Southern Medical University, Guangzhou, 510515 China

**Keywords:** Treg cells, Muscle injury, Muscle regeneration, Inflammation, Macrophage, MuSCs

## Abstract

As the understanding of skeletal muscle inflammation is increasingly clarified, the role of Treg cells in the treatment of skeletal muscle diseases has attracted more attention in recent years. A consensus has been reached that the regulation of Treg cells is the key to completing the switch of inflammation and repair of skeletal muscle, whose presence directly determine the repairing quality of the injured skeletal muscle. However, the functioning process of Treg cells remains unreported, thereby making it necessary to summarize the current role of Treg cells in skeletal muscle. In this review, the characteristics, origins, and cellular kinetics of these Treg cells are firstly described; Then, the relationship between Treg cells and muscle satellite cells (MuSCs), conventional T cells (Tconv) is discussed (the former is involved in the entire repair and regeneration process, while the latter matters considerably in causing most skeletal muscle autoimmune diseases); Next, focus is placed on the control of Treg cells on the phenotypic switch of macrophages, which is the key to the switch of inflammation; Finally, factors regulating the functional process of Treg cells are analyzed, and a regulatory network centered on Treg cells is summarized. The present study summarizes the cell-mediated interactions in skeletal muscle repair over the past decade, and elucidates the central role of regulatory T cells in this process, so that other researchers can more quickly and comprehensively understand the development and direction of this very field. It is believed that the hereby proposed viewpoints and problems can provide fresh visions for the latecomers.

## Background

Treg cells constitute an immunosuppressive subpopulation of CD4^+^ T cells, accounting for approximately 10% of peripheral CD4^+^ T cells in the blood of healthy individuals [1]. In addition to important characteristic markers Forkhead/winged-helix transcription factor 3(Foxp3) and CD25, Treg cells also express co-inhibitory molecules, such as PD-1 and CTLA-4, and co-stimulatory molecules, such as CD134, CD137 and ICOS. The presence of these molecules is the basis for the activation and function realization of Treg cells [2]. Treg cells were initially found in lymphoid tissues, with Treg cells in non-lymphoid tissues firstly proposed and studied in 2009. These tissues include skeletal muscle, visceral adipose tissue (VAT), lamina propria of the colon, skin and myocardium [3]. The focus of the present study was merely on Treg cells in skeletal muscle.

In order to investigate skeletal muscle disease, firstly we should comprehend the process of cellular kinetics after skeletal muscle injury.

When skeletal muscle suffers an acute injury, the destruction of blood vessels will lead to the formation of anaphylatoxins, and trigger the degranulation process of resident mast cells. Destruction of muscle fibers liberates intracellular proteins and molecules normally sequestered in the extracellular matrix, and activates the complement system and other resident cells (including resident neutrophils in muscle, etc.) [4, 7]. C3a and C5a produced after complement system activation [8] and pro-inflammatory molecules produced after the activation of resident cells, including TNF- α, IL- 1 and histamine [4] will bring about more mast cells, neutrophils and other immune cells to be recruited to the injury site. The above multi-channel cell recruitment enables neutrophils to reach the injury site within a few minutes after injury, peak at 6–24 h, and decline rapidly 72–96 h after injury [9]. Neutrophils phagocytose cell debris cleans the injured area and releases enzymes, cytokines and oxidative factors to facilitate the elimination of necrotic muscle. In addition, neutrophils at the injury site produce IL-1 and IL-8, which contributes to the infiltration of monocytes into the lesion site [4, 7].

**Fig. 1:**
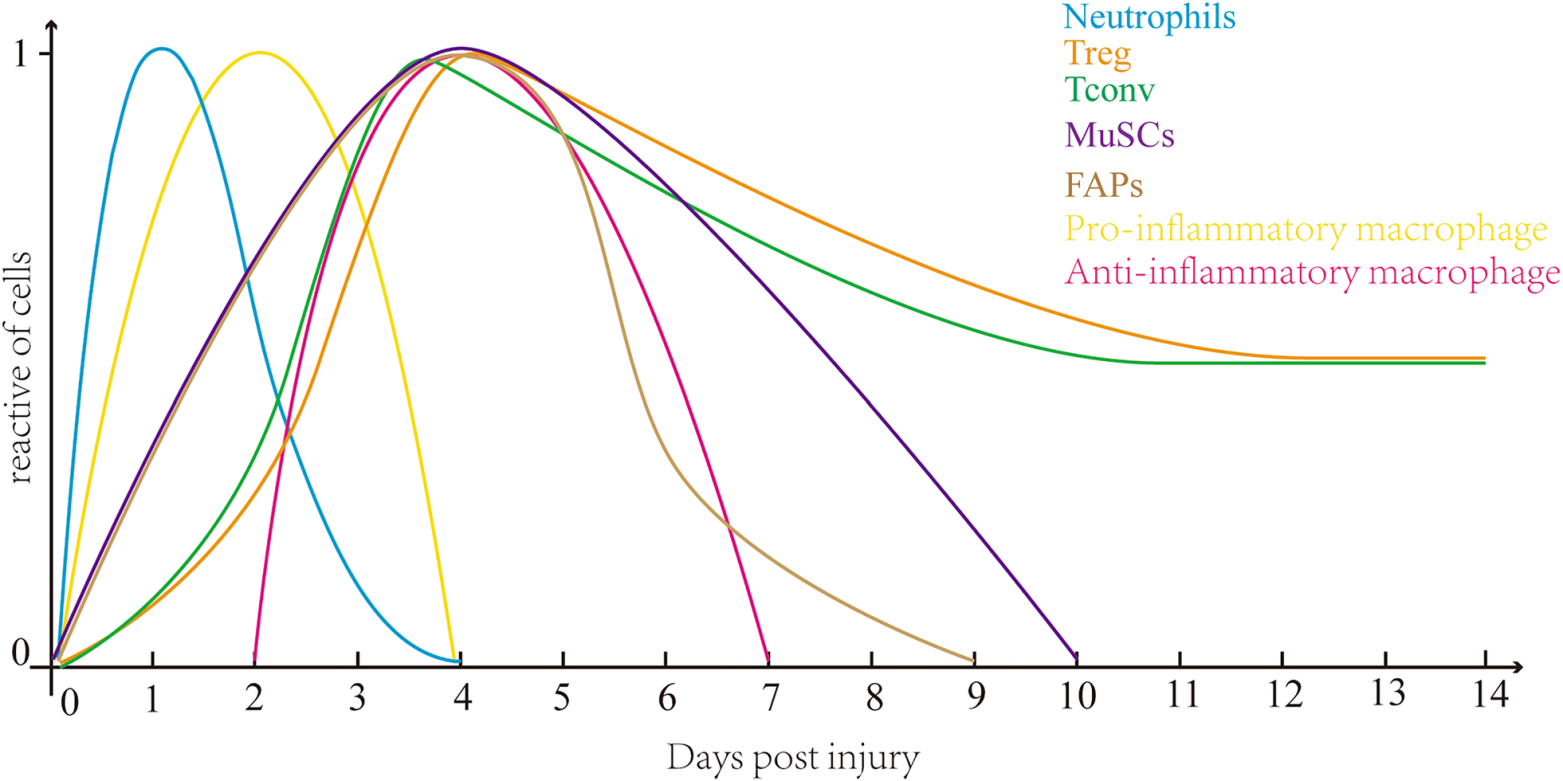
Cellular kinetics after the injury of skeletal muscle. Neutrophils appear on Day 0, peak on Day 1, and disappear on Day 4; Treg appear on Day 0, peak on Day 4, and decrease to half the peak on Day 14; Tconv appear on Day 0, peak on Day 3 or 4, and decrease to half the peak on Day 14; MuSCs appear on Day 0, peak on Day 4, and disappear on Day 10; FAPs appear on Day 0, peak on Day 4, and disappear on Day 9; Pro-inflammatory macrophages appear on Day 0, peak on Day 2, and disappear on Day 4; Anti-inflammatory macrophages appear on Day 2, peak on Day 4, and disappear on Day 7

Over the next 48 h [4], pro-inflammatory macrophages differentiated from monocytes proliferates in the damaged muscle tissue with the decrease of the count of neutrophils, and macrophages thereby become the core inflammatory cells. Pro-inflammatory macrophages promote the phagocytosis of necrotic fibers and fragments, maintain inflammation, and activate the differentiation of MuSCs and the proliferation of myoblasts [10]. Tconv make their appearance about 3 days after injury [4, 11] and play a role in clearing antigens and secreting cytokines, further creating a clean microenvironment for the subsequent regeneration of skeletal muscle. However, the inflammatory process does not last constantly, and the number of Treg cells reaches its maximum about 4 days after injury [11], during which, pro-inflammatory macrophages are replaced by anti-inflammatory macrophages [5], marking the beginning of the anti-inflammatory and muscle regeneration phase. Changes in the number of each cell are shown in Fig. 1.

After the above processes, the relative abundance of Tconv, neutrophils and pro-inflammatory macrophages gradually decreases to a minimum, while that of anti-inflammatory macrophages, fibro-adipogenic progenitors (FAPs) and MuSCs gradually reaches its maximum [12], among which, the presence of FAPs promotes the survival of Tregs, accelerates the transition of stage, and generates new matrix components as a scaffold guiding the generation of new muscle [13]. MuSCs, the main cells in the regeneration stage, differentiate into myoblasts and proliferate in the inflammatory stage. Then, in the regeneration stage, the existence of anti-inflammatory macrophages can strongly stimulate the fusion of myoblasts into major myotubes and complete the subsequent differentiation toward skeletal muscle [14].

It can be found from the cellular kinetics of repair after skeletal muscle injury that regulatory CD4^+^ T cells (Treg cells) expressing Foxp3 play a crucial role in the shift in cellular response. Macrophages and conventional T cells not inhibited during the inflammatory phase can bring about many autoimmune myositis such as idiopathic inflammatory myopathy (IIM), and muscular dystrophy can also be caused by excessive production of Tregs cells and prolonged anti-inflammatory and regenerative stages [15]. Therefore, in order to clarify the role of Tregs cells in the regulatory network of skeletal muscle inflammation, this paper will review recent literature on the relationship of Tregs cells with muscle injury inflammation and repair.

### Special Treg in skeletal muscle

There are many kinds of Treg cells in human body, but CD4^+^ CD25^+^ Foxp3^+^ Treg cells are closely related to immune and inflammatory responses and have been most frequently studied [16]. The most crucial factor that distinguishes Treg cells from other T cells is Foxp3, a member of the Forkhead family of transcription factors, which matters considerably for the development of Treg cells and the formation of immunosuppressive functions. Foxp3 activates genes including CD25 and CTLA-4 [17] on the one hand, while on the other, it inhibits the function of nuclear factor of activated T cells (NFAT) and nuclear factor kappa B (NFκB), and leads to the suppression of genes including IL-2 and T-cell cytokines [17]. Disruption of the Foxp3 gene can cause early onset and fatal multi-organ inflammation and autoimmune diseases [18] associated with skeletal muscle, including idiopathic inflammatory myopathy (IIM) [19] and myasthenia gravis (MG) [20], etc.

Significantly, Treg cells in injured skeletal muscle are a special subset in the CD4^+^ CD25^+^ Foxp3 ^+^ Treg cells population, the prevalence, transcriptome, and T cell receptor (TCR) repertoire of which are different from those in lymphatic organs [11]. In the acute muscle injury model injected with cardiotoxin (CTX) in mice, compared with spleen, CCR2, KLRD1(an activation marker), Amphiregulin (Areg) and IL1RL1(encode ST2 receptor) were highly expressed by Treg cells [11], and Treg cells found in chronic muscle injury models of mdx mice also fell into this category [11]. New work by Burzyn et al. [21] provided a new optimized experimental protocol for isolating and analyzing these Treg cells from damaged skeletal muscle. Indeed, in addition to skeletal muscle, Treg cells also present a diversity in different tissues and organs [22, 23], and their transcriptome expressions are not identical. For example, visceral adipose tissue Treg cells upregulate molecules involved in lipid metabolism (such as LDLR, DGAT, CD36) and the circadian rhythm (such as NR1D1, RORα). Peculiarly, as opposed to skeletal muscle and colon, PPARγ, which regulates adipose differentiation, is also highly expressed [24]. The colonic Treg cells are largely developed against microbial antigens, and are therefore provided with a more enormous TCR repertoire [25]. Meanwhile, recent studies have found that ST2/IL-33 signaling in Treg cells can ameliorate colonic tissue injury and colitis symptoms [26], and this regulatory axis is also observed in skeletal muscle, which will be discussed below.

As for the source of these different Treg cells, they are indeed diversified in the origins, and can be generally divided into thymus derived Treg cells (tTreg cell), peripheral Treg cells generated by Foxp3-T cells (pTreg cell) and induced Treg cells (iTreg cell) in vitro [1, 27]. All Treg cells isolated from injured muscles express Helios transcription factor and neuropilin-1 (NRP1) [11], which is considered a unique marker of tTreg cells different from pTreg cells [28]. To this end, Treg cells in muscle are believed to derive from the thymus into peripheral blood, and are recruited to the injury site from peripheral blood to play their role. However, when and where muscle Treg cells undergo transcriptome and other changes that differentiate them from other tTreg cells remains unreported.

In terms of cellular kinetics, after acute injury, the number of Treg cells begins to rise on the first day and reaches a peak around the fourth day. After that, the number of Treg cells decreases gradually but remains at a low level for at least 30 days [11]. As was mentioned earlier, the time when the number of Treg cells reached the peak is rather close to the peak of the number of anti-inflammatory macrophages, MuSCs and fibro-adipogenic precursor cells, while the number of neutrophils and pro-inflammatory macrophages reached their troughs [12]. This quantitative relationship corresponds to the relationship between Treg cells and other cells below. Although the timing and role of various cells in acute injury are well understood, chronic muscle injury is still unclear. Experiments have shown that the number of Treg cells in the muscle of mdx mice is increased [11], even more than that in acute injury. The role of Treg cells at this point is to reduce skeletal muscle damage and inhibit fibrosis, which can be achieved by secreting cytokine IL-10, down-regulating genes that cause muscle fibrosis such as osteopontin (SPP1) and connective tissue growth factor (CTGF) [11], and inhibiting the expression of IFN-γ, Granzyme B, PD-1 and CXCR3 [29, 30]. Besides, the infiltration of anti-inflammatory macrophages can be observed in mdx mice at the early necrosis at the age of 4 weeks of age [31], and the early increase in the number of macrophages may be connected with the function of Treg cells to induce the phenotype switch of macrophages.

### Regulation of MuSCs by Treg cells

MuSCs, also known as muscle stem cells, are mainly derived from Pax3^+^/ Pax7^+^ embryonic progenitor cells [32, 33], the typical biomarker on the surface of which is Pax7 [34, 35], that plays a key role in maintaining the fixation and proliferation of progenitor cells and preventing early differentiation and apoptosis [34]. The combination of Pax7 with FACS is sufficient to effectively separate MuSCs from muscle tissues [36], while the absence of Pax7 will lead to impaired repair of damaged skeletal muscle, aggravation of fibrosis and massive deposition of adipose tissue [38]. MuSCs are heterogeneous. Previous studies have shown that there are at least two different types of cells in MuSCs, including Pax7 ^hi^ Myf5^−^ stem cells, which maintain the MuSCs cell pool, and Pax7 ^lo^ Myf5^+^ myogenic progenitor cells [41] that will continue to differentiate into Myf5^+^ myoblasts [39, 40] for skeletal muscle repair.

It makes sense to link Treg cells and MuSCs together. In fact, some studies followed histologically the presence of Treg cells near the grew again fibers after skeletal muscle injury, and the muscle rich in Treg cells is equipped with a large MuSCs bank [11, 45]. Current studies have indicated that Treg cells regulate MuSCs mostly through Areg [11, 45, 46], a member of the epidermal growth factor (EGF) family that stimulates the proliferation of most cell types. Such an effect is mainly mediated by binding and activating the widely expressed transmembrane tyrosine kinase epidermal growth factor receptor (EGFR) and the downstream signaling pathways including Ras/MAPK, PI3K/Akt, mTOR, STAT, and PLCγ [47]. Areg added to mice with deficient Treg cells for therapy can improve the differentiation of MuSCs [48] and the expression of highly expressed genes in healthy muscles (such as Pfkfb1and Myl2), inhibit fibrosis-related genes (such as Acta2, Adam12), and normalize muscle transcriptome [11, 49].

Areg is expressed not only by Treg cells, but also by various immune cells, including mast cells, eosinophils, basophils, ILC2 and dendritic cells under different inflammatory stimuli [50]. This feature of Areg in multiple immune cells leads to an interesting result that Treg cells secrete Areg to regulate MuSCs while receiving Areg secretion from other immune cells. For example, mast cells are observed in chronic inflammation, while basophils are present in acute inflammation [50]. EGFR is upregulated through STAT5 signal transduction after the activation of Treg cells. The inhibition ability of Treg cells after binding to Areg may be improved by secreting exosomes containing immunosuppressive RNA [51], while Treg cells without Areg will fail to exercise their function of inhibiting local inflammation [50], but the reason remains unclear.

In addition, Treg cells can secrete the inhibitory factor TGF-β [52]. It has been found that macrophage-derived Areg can bind to the ligand EGFR on the surface of pericytes, and then activate the integrin-αVβ, which will then activate and drive the differentiation of pericytes into myofibroblasts through the TGF-β signaling pathway[6], thereby promoting secretion of extracellular matrix (ECM) and accelerating wound healing [53]. To this end, if the Areg secreted by Treg cells is also provided with the same function, a hypothesis can be proposed that after skeletal muscle injury, Areg and TGF-β secreted by Treg cells will move towards two directions, one to form muscle fibers, and the other to form the matrix among muscle fibers, which will jointly complete the repair process of skeletal muscle., it also has been reported that TGF-β can stimulate the production of Areg in lung fibroblasts [54], but whether this can be accomplished in MuSCs needs further validation.

### Treg cells and Tconv

Tconv are involved in a wide range of skeletal muscle diseases, including acute muscle injury [11, 55, 56], IIM [57], myasthenia gravis [58] and chronic muscular dystrophy [59].

If the anterior tibial muscle of exogenous ovalbumin (OVA)-injected mice is mechanically compressed, the OVA can act as a foreign soluble antigen to induce an immune response, and leads to the increase in the numbers of CD4^+^ and CD8^+^ Tconv [55]. The same phenomenon can also be found in the muscle of mice injected with CTX [11]. Indeed, the infiltration of such Tconv in skeletal muscle is necessarily important. Zhang et al. [56] found that CTX injection in mice lacking CD8^+^ Tconv will lead to decreased ability of fiber regeneration and increased matrix deposit. This is attributed to the missing of CD8^+^ Tconv, which will result in the deficiency of MCP-1 and then the reduced recruitment of Grl^hi^ macrophages. Besides, Fu et al. [60] claimed that IL-1α, IL-13, TNF-α and IFN-γ secreted by Tconv can promote the proliferation of MuSCs. These results indicate that Tconv can create a favorable microenvironment for the growth of muscle cells by eliminating antigens and secreting cytokines during acute muscle injury. However, if Treg cells are depleted, the total number of Tconv in the acutely injured skeletal muscle will be significantly increased, and many Tconv-specific genes will be overexpressed, thus leading to increased fibrosis and collagen accumulation [11].

Targeting at autoantigens, Tconv are involved in the pathological process of IIM, including polymyositis (PM), dermatomyositis (DM) and inclusion body myositis (IBM). CD8^+^ Tconv play an important role in the pathogenesis of PM and IBM, while CD4^+^ Tconv are more closely related to DM [57]. CD8^+^ Tconv in PM and IBM serve to bind muscle fibers bearing the MHCI marker and destroy the sarcolemma [61], while CD4^+^ Tconv in DMare induced to differentiate into Th1, Th2 and Th17 cells, among which, Th1 cells can induce the production of pro-inflammatory macrophages by producing large amounts of IFN-γ and TNF-α, and IL-4 produced by Th2 cells is mainly related to the production of IgE and the recruitment of eosinophils [61]. Th17 cells produce IL-17 and bind to IL-1, so that myoblast cells can produce IL-6, MHCI and chemokine CCL20 [61, 66]. Such Tconv-mediated autoimmunity in IIM is largely attributed to the significant decrease in the number of Treg cells [66]. In DM, Treg cells from peripheral blood are characterized by the low CTLA-4 expression and the impaired function [67], whereas in PM and IBM models, the addition of Treg cells in vitro reduces CD8 ^+^ T cell myolysis [68].

For MG, it is Tconv that lead to the production of pathogenic autoantibodies, mainly anti-AChRs that bind to AChRs at the neuromuscular junction (NMJ) and induce the complement to produce the attack complex C5B6789n, thereby resulting in NMJ injury, the production of inflammatory cytokines (including TNF-α, IL-6, and IL-1β) and the subsequent decrease in muscle function. The occurrence of MG is considered relevant to the regulatory dysfunction of Treg cells, which is further related to the down-regulation of CTLA-4, Foxp3 and IL-10 [58].

CD8^+^ Tconv are also involved in chronic muscular dystrophy [59]. Recent studies have confirmed the decrease in the number of CD8/CD26 Tconv in the peripheral blood of patients with DMD [69]. The CDR3 region of TCR Vβ2 expressed by these Tconv possesses a conserved sequence of four amino acids, suggesting the specific antigen recognized by Tconv in inflammatory infiltration [70], which is not a common feature of diseases concerning muscle tissue inflammation. Meanwhile, all DMD muscle fibers invaded by CD8 + Tconv express MHC class I molecules on their surface [71], and these cells, once activated, can migrate and recognize specific peptides on the surface of muscle fibers triggering the release of perforin, granzyme and TNF-α that damages muscle cells.

In conclusion, Tconv play a dual role in skeletal muscle: on the one hand, they clear up antigens for other cells, adjust the microenvironment and facilitate the repair, while on the other, they act as a driving force for muscle injury when the immune system is disordered, highlighting the particularly important role of Treg cells that regulate the function of Tconv.

The ways that Treg cells regulate Tconv include direct and indirect inhibition. IL-2 is involved in the direct inhibitory effect. The antigen-induced IL-2 produced by Tconv can complete its own proliferation in the form of autocrine. At the same time, IL-2 is also an indispensable cytokine for Treg cells to obtain the expression of Foxp3 [30], while the marker CD25 on the surface of Treg cells is actually the α chain of IL2-R, which indicates that Treg cells can consume IL-2, thereby inhibiting Tconv activation and proliferation. Interestingly, this regulation exercises considerable effect on CD8^+^ Tconv, but limited effect on CD4^+^ Tconv [72]. Benveniste has proven that the inhibition of Tregs can accelerate the progression of the disease, while Treg injections can ward off the disease and reduce CD8^+^ Tconv penetration in IBM [73].

In addition, the activation of Tconv is also regulated by some indirect approaches, the most obvious of which is CTLA-4 that traps B7 molecules (CD80 and CD86) on DCs and thus prevents them from binding to CD28 on Tconv [74]. CTLA-4 can also induce the upregulation of indoleamine 2, 3-dioxygenase (IDO), a tryptophan breakdown enzyme on DCs, thereby inducing its regulatory phenotype [75]. Furthermore, Treg cells can express CD39 and CD73 to catalyze the degradation of nucleotides, such as ATP, into adenosine that inhibits the proliferation of activated CD4^+^ Tconv by binding to the A2A receptor [76, 77], which is beneficial for the reduction of inflammation.

### The key to the phenotypic switch of macrophages: Treg cells

Next, the most important role in skeletal muscle inflammation, macrophages are to be introduced. Normally, there are few macrophages in skeletal muscle, and the resident macrophages are mainly in the connective tissue surrounding the muscle bundles [78, 79]. When skeletal muscle is injured, monocytes/macrophages are recruited from the blood to function at the injury site. Macrophages can be divided into pro-inflammatory and anti-inflammatory cells according to the different polarization,

Macrophage polarization is regulated by a variety of factors. Inflammatory components such as LPS and IL-12, and Th1 cytokines such as IFN-γ and TNF-α can polarize monocytes into pro-inflammatory macrophages [59, 80] that are associated with the high expression of iNOS, production of reactive oxygen and nitrogen, secretion of TNF-α, IL-8, IL-1 β, IL-6 and IFN-γ, and production of growth factors VEGF and FGF2 [81, 82]. Pro-inflammatory macrophages can promote tissue repair and affect the phagocytosis of damaged/necrotic muscle fibers as well [78]. However, excessive production of pro-inflammatory macrophages may lead to tissue damage and autoimmune diseases [83]. Muscle loss caused by decreased function is closely related to the prolonged pro-inflammatory response and high fibrosis of new tissues [84]. Immune complexes, glucocorticoids and cytokines such as IL-4, IL-13 and IL-10 can induce the production of anti-inflammatory macrophages [59] that regulate the immune response mainly by secreting IL-4, IL-10 and TGF-β, control the inflammatory response, and promote the formation of blood vessels, new muscle fibers and tissue remodeling [78, 82, 85].

The above statement has fully manifested the essential role of macrophages in the inflammation of damaged skeletal muscles, the phenotypic switch of which is necessary to complete the whole repair process, with Treg cells exactly serving as the key to the control over that. In a mouse skeletal muscle inflammatory model built with CTX, Treg cells rapidly increase during the period when monocytes switch from a pro-inflammatory cell population (Ly6C^+^) to an anti-inflammatory cell population (Ly6C^-^) [11], suggesting that Treg cells can influence the pro-inflammatory to anti-inflammatory switch of monocytes.

In previous studies, the increased expression of CD206, CD47, CD23, TLR2, TLR4, argininase activity and IL-10 were detected, and phagocytosis of macrophage was enhanced after CD4^+^ CD25^+^ Treg cells were directly injected into the peritoneal cavity of SCID (T and B cell-deficient) mice [88], indicating the role of CD4^+^CD25^+^ Treg cells in effectively inducing the production of anti-inflammatory macrophages. Similarly, in skeletal muscle injury model, the key point of Treg cells regulating macrophages is to control the phenotypic switch, including inhibiting the production of pro-inflammatory macrophages and promoting the production of anti-inflammatory macrophages.

In the second part, it was mentioned that Treg cells in skeletal muscle can inhibit the activation and proliferation of Tconv [89]. In fact, Treg cells can indirectly regulate macrophages in this way. After muscle injury, infiltrating CD8^+^ Tconv promote CCL2 secretion, and lead to recruitment of pro-inflammatory macrophages [56]. By inhibiting CD8^+^ Tconv, Treg cells can indirectly reduce the recruitment of pro-inflammatory macrophages and avoid excessive inflammation. A recent study has identified a regulatory layer of IFN-γ, which induces the production of pro-inflammatory macrophages. Treg cells reduce the activation of MHC Class II Transactivator(CIITA)by inhibiting excessive IFN-γ production of NK and Tconv, thereby reducing the expression of MHC II in macrophages, i.e., reducing the numbers of pro-inflammatory macrophages [90]. Besides, experiments in mdx mice have justified the same conclusion that the depletion of Treg cells can enhance IFN-γ response, activate pro-inflammatory macrophages [29], and aggravate muscle injury. The discovery of this regulatory layer provides a pathway for the muscle immunotherapy of NK cells and macrophages regulated by Treg cells through IFN-γ. Recent studies have proven that CaMKIV is upregulated in CTX-damaged skeletal muscle, which results in the decrease of Treg cells in skeletal muscle [91]. Knocking down CaMKIV attenuates IFN-γ-induced IL-1β, IL-6, and TNF-α in the myotube, but upregulates the MIP-1α and MCP-1 [92]. It can then be reasonably inferred that knocking down CaMKIV may reduce the number of pro-inflammatory macrophages through the IFN-γ regulatory layer by increasing the number of Treg cells. Treg cells can affect tissue regeneration by secreting several immunosuppressive cytokines and regulating local inflammation after injury. These cytokines include TGF-β, IL-10 and IL-35, among which IL-10 and TGF-β can induce the polarization of anti-inflammatory macrophages [76, 86]. Moreover, such a process can be accelerated by the rapid accumulation of Treg cells [93]. Treg cells can also induce the expression of molecules similar to heme oxygenase 1 (HMOX1) in target tissue cells [59], and the lack of HMOX1 secreted by macrophages will cause damage to skeletal muscles [87, 94]. The regulation of HMOX1 production induced by Treg cells is believed to be related to the macrophage polarization, but the specific mechanism is still unclear.

Model or type of skeletal muscle injury affects the functions of Treg cells. For example, Jin et al. [95] found in the chronic toxoplasma gondii skeletal muscle infection model that Treg cells could increase the production of pro-inflammatory macrophages and aggravate skeletal muscle injury, while the ablation of Treg cells instead saved macrophage homeostasis and skeletal muscle fiber regeneration [96]. However, with the supplementation of Treg cells derived factors such as IL-10 and Areg, the macrophage phenotype was biased towards anti-inflammatory type, which may result from the qualitative change of Treg cells. Similarly, gender also affects skeletal muscle repair. Recent studies have showed that estrogen can promote the switch of macrophages from pro-inflammatory type to anti-inflammatory type, and reduce the levels of inflammatory factors IL-1β, SLPI and MCP-1 [97]. The number of Treg cells increase in the high estrogen state, when the Th cells will differentiate into the Th2 phenotype, suggesting that estrogen may inhibit the production of pro-inflammatory macrophages in the Th1 response by increasing the number of Treg cells and Th2 response [98].

On the contrary, macrophages can also regulate Treg cells. Recent studies have indicated that the effectiveness of glutamine (GLN) is inhibited after muscle injury, that low levels of GLN stimulates the secretion and oxidation of GLN by infiltrating macrophages, and that GLN released by macrophages can activate mTOR and promote the proliferation and differentiation of MuSCs after the uptake by MuSCs [99]. Another study has proposed that GLN might up-regulate the number of Treg cells through the IL-2 and IL-2 receptor pathways, and similarly, the administration of GLN can reduce the recruitment of pro-inflammatory macrophages and increase the polarization to anti-inflammatory macrophages [15]. It can be speculated from the above investigations that there is a negative feedback system in macrophages, and GLN can be an important factor involved in this system. The low level of GLN in the injured skeletal muscle stimulates the macrophages secreting GLN. GLN secreted by macrophages, on the one hand, promotes the repair of the injured skeletal muscle while on the other hand, guides the switch from pro-inflammatory to anti-inflammatory. Besides, Treg cells are likely to be enhanced by GLN and play an important role in this system.

New studies have shown that programmed cell death protein 1 (PD-1), also known as CD279, can promote the regeneration of contused skeletal muscle by regulating Treg cells production and macrophage polarization [100]. PD-1, belonging to the CD28 / CTLA-4 / ICOS co-stimulatory receptor family, was discovered in mouse T-cell hybridoma cell lines by Japanese scientist Ishida in 1992 [101], which is involved in inhibiting T and B cell proliferation and cytokine production through its specific ligands B7-H1 (PD-L1/CD274) or B7-DC (PD-L2) [102], and can be expressed on activated T cells, B cells, NK cells, monocytes and DC cells. It is also widely expressed in non-blood cells and tumor cells [103]. The PD-1/PD-L1 axis plays an important role in the development and function of Treg cells. To be specific, PD-L1 can directly induce the transformation of Treg cells in vitro, or increase the Foxp3 expression on Treg cells and enhance the immunosuppressive ability of Treg cells. In addition, PD-L1 can also transform primitive CD4^+^ T cells into Treg cells by down-regulating Akt, mTOR and ERK2 and simultaneously up-regulating PTEN [104]. PD-1 can induce the expansion of Treg cells by regulating the Notch pathway, or stabilize Foxp3 on Treg cells by inactivating asparaginyl endopeptidase (AEP) [105]. Therefore, for skeletal muscle, the number of Treg cells, the secreted Areg, IL-4 and IL-10 secreted by macrophages is decreased after PD-1 knockout, while that of pro-inflammatory macrophages and the expressions of inflammatory factors IL-1β, IL-6 and TNF-α is increased [100]. Furthermore, the depletion of Treg cells in mdx mice will indirectly lead to a significant increase in the expression of PD-L1 on pro-inflammatory and anti-inflammatory macrophages and a significant decrease in the expression of anti-inflammatory -activated marker CD206, but will exercise no effect on pro-inflammatory macrophages [29]. However, whether this means that anti-inflammatory -type macrophages need PD-1 signal on Treg cells to realize phenotypic transformation, that is, whether Treg cells regulate phenotypic transformation of macrophages by means of the PD-1/PD-L1 axis still needs further verification.

### Various factors regulating the function of Treg cells in skeletal muscle inflammation

#### FAPs promote the survival of Treg cells with the help of IL-33

There is direct immunofluorescence evidence proving that IL-33-expressing cells and Foxp3^+^ expressing Treg cells clusters are close to and partially overlapping after muscle injury [106], suggesting that the accumulation of Treg cells is regulated by IL-33 produced by a population of mesenchymal stem cells, known as FAPs. IL-33 is a member of the IL-1 family of cytokines, which, as an alarm protein, is stored in the nucleus and acts as a cytokine released extracellularly in response to cell or tissue damage [107]. The receptor of IL-33 is ST2, which can almost be expressed in all immune cells [108]. However, it has been found that the proportion of Treg cells expressing ST2 receptor after skeletal muscle injury is much higher than that of other immune cells [45], indicating that the application of IL-33: ST2 axis can specifically enhance the survival of Treg cells. Meanwhile, IL-33 can promote macrophages to express CCR2 and recruit them to the injury site. Besides, IL-33 can also regulate the pro-inflammatory activity of macrophages through Toll-like receptors [107].

The FAPs that secrete IL-33 need to be mentioned for a deeper understanding of this mechanism, which are mesenchymal progenitor cells in skeletal muscle, provided with the ability of stem cells to differentiate into adipocytes and fibroblasts, characterized by specific cell surface markers CD45^-^ CD31^-^ SCA1^+^ PDGFRA^+^ [86], and usually surrounded by a large number of neural structures, indicating that IL-33 secreted by FAPs can transmit signals between the nervous system and the immune system in the muscular environment [45]. In fact, Wang et al. [106] have identified that FAPs not only secrete IL-33, but also have a receptor for calcitonin gene-related peptide (CGRP). CGRP functions in pain transmission in the nervous system, suggesting that pain after skeletal muscle injury can indirectly increase the recruitment of Treg cells through CGRP signals. It can therefore be inferred that there may be a neuro-mesenchymal stem cell-immune network involved in skeletal muscle repair.

FAPs, macrophages, MuSCs and Treg cells can jointly form an intercellular regulatory network. After injury, neutrophils arrive at the injury site firstly, followed by pro-inflammatory macrophages. In order to prevent FAPs from differentiating into fibroblasts and adipocytes in the environment where necrotic tissue is not completely cleared, pro-inflammatory macrophages at this period will secrete TNF-α and promote apoptosis of FAPs [109]. However, the subsequent anti-inflammatory macrophages in turn promote the proliferation of FAPs by secreting TGF-β [109]. The important role of Treg cells in the phenotypic switch of macrophages endows this step with great significance. The increase of Treg cells starting from the first day can induce the generation of anti-inflammatory macrophages to proliferate FAPs, resulting in increased IL-33 secreted by FAPs. If the process of macrophages regulating FAPs and Treg cells regulating macrophages cannot be carried out normally, FAPs will eventually promote the production of fibrosis by secreting type I and III collagen and connective tissue growth factor (CTGF) [110], which can be observed in the experiment of mdx mice, and may be the pathological basis of muscular dystrophy [15, 44]. MuSCs are the effector cells in the repair process of skeletal muscle and are regulated by the above three types of cells in a staged manner [12, 111]. FAPS cells and pro-inflammatory macrophages affect the differentiation of MuSCs into myoblasts and their proliferative stage through IGF-1, IL-6, Wnt1, Wnt3A ,Wnt5A [112], WISP1 [113], follistatin [114], IL-10 [115], IL-1β and IL-6, HGF, IGF-1, VEGF and high concentrations of TNF-α [59, 78, 81], respectively. Afterwards, anti-inflammatory macrophages and Treg cells affect the stage of myoblast differentiation into myotubes through TGF-β, IGF-1, low concentration of TNF-α [78], and Areg [11], respectively.

### Specific TCRs on Treg cells

From the conventional immune perspective, the focus of present research is shifted to another regulatory path. Burzyn et al. [11] isolated the TCR stockpile from many damaged skeletal muscles of mice in multiple clones. In general, TCRs are rarely the same, except one named mTreg24, which is co-expressed in most mice. Cho et al. [116] constructed a transgenic mouse strain expressing mTreg24 using the primers for this clone, and have found that Treg cells in this strain of mice are more rapidly aggregated after CTX-induced skeletal muscle injury than those in the control group, suggesting that antigen recognition by specific TCR may be one of the driving forces of Treg cells aggregation. However, the doubt still exists whether this specific TCR can be applied to other injuries. Cho et al. transferred mTreg24 into mdx mouse models and have found that muscle regeneration is enhanced [116], indicating that the enhancement of TCR may be a target for the treatment of skeletal muscle diseases, but further studies are still needed to test the therapeutic effect of mTreg24 in a variety of models.

### The presence of ATP/P2X axis inhibits the recruitment of Treg cells

The muscle membrane will rupture after the injury of skeletal muscle, when the energy-supplying ATP will be released into the tissue interval in large quantities, turning into a molecular pattern molecule (DAMPs) [117]. The presence of high concentrations of ATP activates a nonselective cationic channel called P2 purinergic receptor (P2XR) on cells, especially the P2 × 7R subtype [118]. ATP triggers K^+^ outflow while binding to P2 × 7R, when inflammasomes NLRP3 will be activated and IL-1β will be released, thereby resulting in the release of downstream inflammatory mediators [119]. Normally, high expression of P2 × 7R on myoblast can stimulate their own proliferation and complete the differentiation towards myotubes [120]. However, once skeletal muscle enters a pathological state, the inflammatory environment facilitated by the ATP/P2 × 7R axis will accelerate the death of muscle cells [120]. Undoubtedly, this inflammatory environment is rather hostile to anti-inflammatory Treg cells. Existing studies have shown that ATP can inhibit the function of Treg cells with the help of P2 × 7R, which might be achieved by reducing the abundance of Foxp3 protein, transforming Treg cells into Th17 cells or activating the ERK pathway [121], which makes the removal of P2 × 7R beneficial to skeletal muscle repair. Some experiments have confirmed the existence of high P2 × 7R expression in mdx mice. If P2 × 7R gene is knocked out in mdx mice, the infiltrating leukocytes in muscle will be reduced, while the number of Treg cells will be increased instead [122], indicating that the inhibition of ATP/P2X axis can increase the number of Treg cells. Unfortunately, this phenomenon is found only in models of muscular dystrophy and can be used as a potential treatment for this disease, but there still lacks research on acute injury model.

## Conclusions

**Fig. 2:**
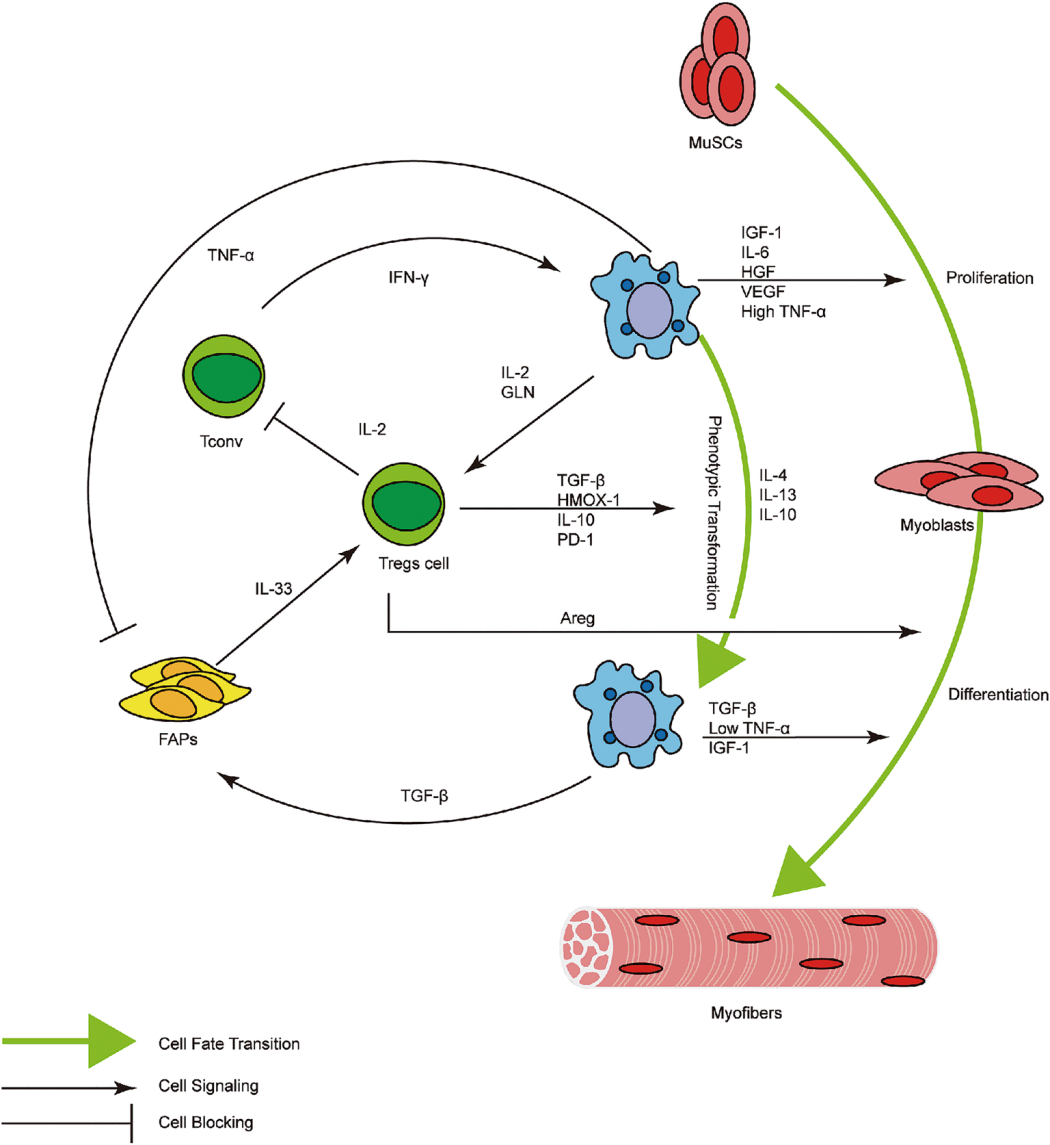
Schematic summary of the Treg-centered regulatory system in skeletal muscle repair. Treg cells can secrete IL-2 to inhibit Tconv and indirectly inhibit pro-inflammatory macrophages; they are stimulated by IL-2 and GLN secreted by pro-inflammatory macrophages, and in turn secrete TGF-β, HMOX-1, IL-10 and PD-1 for completing the phenotype switch of macrophages; Treg cells are also stimulated by IL-33 secreted by FAPs, which is inhibited by TNF-α secreted by pro-inflammatory macrophages and promoted by TGF-β secreted by anti-inflammatory macrophages; besides, Treg cells can directly secrete Areg or indirectly control the proliferation and differentiation of MuSCs by affecting the phenotype switch of macrophages.

In summary, this paper reviewed the role of Treg cells in skeletal muscle inflammation and regeneration, also the current regulatory mechanism of Treg cells in this process, which takes into account the function of Treg cells in the whole process of regulating inflammation progression, repair and regeneration, goes beyond the previous pattern that Treg cells only target T cells, and fully demonstrates how Treg cells inhibit pro-inflammatory cells such as Tconv and pro-inflammatory macrophages and how they promote the function and effect of anti-inflammatory macrophages, MuSCs and others. All these relationships are shown in Fig. 2.

Specifically, the most important part in the case of Tconv is to summarize the destructive effect of these cells in the pathogenesis for acute and chronic skeletal muscle diseases, and the important reason of their immune dysregulation is the absolute or relative decrease of Treg cells. However, the actual use of Treg cells injection for solving Tconv in these diseases is in the stage of animal experiment, and more experiments should be conducted to prove the applicability of this method to humans. At the same time, some researchers have proposed that Tconv and Treg cells can be transformed into each other. There still exists a doubt whether this means that our research should go deeper into a more fundamental mechanism regulating the differentiation of these two types of cells. The repair and regeneration of skeletal muscle disease cells should be inevitably explored when it comes to MuSCs, which have often been lumped together with macrophages in previous studies. Different types of macrophages play a positive role in MuSCs during normal inflammation and repair, but excessive strength of either of them will result in immune imbalance and failure of skeletal muscle regeneration. Some studies have found that the number of Treg cells in chronic skeletal muscle diseases is much higher than that in usual diseases. The problem can thus be reasonably proposed whether Treg cells also play a dual role in MuSCs. Exploration on macrophages is the most emphasized part of this review, since unlike other cells, macrophages exhibit a bipolarity in this process (pro-inflammatory and anti-inflammatory), which is common to the process in which inflammation counterbalances pro-inflammatory and anti-inflammatory factors. Just like a balance, one side will break the balance solely, and the imbalance will lead to obstacles in the development and repair of various skeletal muscle diseases. Moreover, the step on the middle of that balance is Treg cells. Even though Treg cells have many ways of acting on macrophages, such as regulating IFN-γ released by T cells and NK cells, directly secreting anti-inflammatory regulatory factors such as IL-10 and TGF-β, etc., and possibly affecting macrophages through the PD-1/PD-L1 axis, they all end up on the phenotypic switch of macrophages. It turns to be a general direction of future researches on the treatment of skeletal muscle diseases to better control this turning point.

The function of Treg cells is rather important, making it necessary to explore how they are affected by other cells. Interestingly, the propose of neural-FAPS-IL-33-Treg cells regulatory axis is to combine the nervous and immune system together, which indicates that the inflammation of the skeletal muscle repair, is likely to be a systemic process, but whether this very process cover the blood or endocrine system, etc. needs further verification. The experiment on whether common TCR (mTreg24) can be used for enhancing the function of Treg cells also offers a new vision to transform Treg cells themselves, when transcriptome and costimulatory molecules can all be potential selection objects. For example, reflections should be made upon what will happen to Treg cells in skeletal muscle as they move from the thymus to the injury site. For the ATP/P2X axis, however, its role in the recruitment of Treg cells has only been demonstrated in the chronic skeletal muscle injury model. Though, theoretically, this result supports the acute injury model, experimental evidence is still needed to confirm such a claim. As mentioned above, Treg cells cannot function without Areg secreted by other immune cells, and for macrophages, GLN can also promote the proliferation of Treg cells through the IL-2/IL-2 receptor pathway. To this end, the question is whether it can be reasonably speculated that all other cells involved in skeletal muscle repair, such as pericytes, vascular endothelial cells, dendritic cells, and B cells, play a role of information presentation in Treg cells. As experiments continue to expand, the vast regulatory network of Treg cells that damage skeletal muscle will be gradually clarified, and an increasing number of tools will be proposed to fight against skeletal muscle diseases.

## Data Availability

All data generated or analysed during this study are included in this published article.

## Abbreviations

ApoE: Apolipoprotein E
Areg: Amphiregulin
DM: Dermatomyositis
DMD: Duchenne muscular dystrophy
FAPs: Fibro-adipogenic progenitors
Foxp3: Forkhead/winged-helix transcription factor 3
IBM: Inclusion body myositis
IFN-γ: Interferon-γ
IIM: Idiopathic inflammatory myopathy
MG: Autoimmune myasthenia gravis
MuSCs: Muscle satellite cells
NFAT: Nuclear factor of activated T cells
NFκB: Nuclear factor kappa B
OVA: Ovalbumin
P2XR: Purinergic p2 receptor
PD-1: Programmed cell death protein 1
PM: Polymyositis
SRB1: Scavenger receptor class B1
Tconv: Conventional T cell
TGF: Transforming growth factor
Treg: Regulatory T cell
